# Systematic Review of Lead Exposure and Its Effects on Caries and Aesthetics in Children and Adolescents

**DOI:** 10.3390/healthcare13121460

**Published:** 2025-06-18

**Authors:** Gianina Tapalaga, Livia Stanga, Ioan Sîrbu

**Affiliations:** 1 Department of Odontotherapy and Endodontics, Faculty of Dental Medicine, “Victor Babes” University of Medicine and Pharmacy Timisoara, Eftimie Murgu Square 2, 300041 Timisoara, Romania; tapalaga.gianina@umft.ro; 2 Discipline of Microbiology, Faculty of Medicine, “Victor Babes” University of Medicine and Pharmacy Timisoara, Eftimie Murgu Square 2, 300041 Timisoara, Romania; 3 Department of Oral Implantology, Faculty of Dental Medicine, University of Medicine and Pharmacy “Carol Davila”, 050474 Bucharest, Romania; office@drsirbu.ro

**Keywords:** lead exposure, dental caries, aesthetics, deciduous teeth, children, systematic review

## Abstract

**Background**: Early childhood dental decay remains a pervasive chronic condition, and environmental toxicants—particularly lead—may exacerbate its development. This systematic review was designed to synthesize evidence on how lead exposure correlates with both the occurrence of carious lesions and aesthetic alterations in children’s primary teeth. **Methods**: A comprehensive search was conducted in PubMed, Scopus, and Web of Science through April 2025, selecting observational investigations that assessed the link between lead levels and primary-tooth decay in pediatric cohorts. Thirteen eligible studies, encompassing 44,846 participants aged 2–19 years, were included for qualitative synthesis. Aesthetics were screened using author-defined enamel-defect or discoloration endpoints; however, only three studies reported compatible metrics, precluding quantitative pooling. Heterogeneity in exposure matrices likewise ruled out meta-analysis. **Results**: Most studies reported a statistically significant association between higher lead burden and greater prevalence or severity of caries in primary teeth. Blood lead concentrations across studies ranged from means of 1.53 μg/dL to geometric means of 7.2 μg/dL. Notably, elevated lead was linked to increased decayed, missing, or filled surfaces—with an adjusted risk ratio of 1.14 (95% CI: 1.02–1.27) at levels below 5 μg/dL—and adjusted mean ratios of up to 2.14 for decayed or filled teeth when blood lead reached 5–10 μg/dL. **Conclusions**: Current evidence suggests that children’s exposure to lead may heighten the risk of caries and detract from the aesthetic quality of primary teeth. However, variability in study design, lead quantification methods, and confounder adjustment limit the consistency of findings. Mitigating lead exposure in early life could represent a valuable preventive strategy against dental decay in susceptible pediatric populations.

## 1. Introduction

According to the WHO Global Oral Health Status Report 2022, ~514 million children worldwide live with untreated caries in primary teeth—almost double the burden in permanent teeth—and the prevalence has remained static for two decades despite preventive programs [1]. Early childhood caries can lead to pain, infection, difficulty in eating and speaking, and can have long-term effects on the permanent dentition [2]. Despite advancements in preventive dentistry, the prevalence of dental caries in deciduous teeth continues to be a major concern, particularly in low-income populations, where prevalence can be as high as 80% [3].

The etiology of dental caries is multifactorial, involving a complex interplay between host factors, dietary habits, oral microbiota, and environmental influences [4]. Among environmental factors, exposure to heavy metals such as lead has garnered attention due to its potential impact on dental health [5]. Lead is a pervasive environmental pollutant that can accumulate in biological tissues, including developing and mature teeth, where it may interfere with normal enamel formation, reduce salivary flow and buffering capacity, and alter oral microbiota composition, disrupt enamel mineralization, reduce salivary flow, alter pH, and impair pellicle formation, collectively weakening oral defenses and promoting cariogenic bacterial growth, all of which contribute to an increased risk of dental caries [5,6].

Children are particularly susceptible to lead exposure due to behaviors like hand-to-mouth activity and a higher gastrointestinal absorption rate than adults, which can lead to greater systemic accumulation and increased risk of adverse effects, including dental caries [7]. Common sources of lead exposure include lead-based paints, contaminated soil and water, and industrial emissions [8]. Lead can be incorporated into developing teeth during enamel formation or can accumulate in teeth post-eruptively [6].

Several studies have investigated the association between lead exposure and dental caries in children, with varying results [9,10]. Some studies have reported a positive correlation between higher lead levels in blood, saliva, or tooth enamel and increased caries incidence or severity [9,10,11,12].

However, the evidence is not entirely consistent, and some studies have found no significant association between lead exposure and dental caries [10,11,12,13,14,15,16,17,18,19,20,21,22,23,24]. Differences in study design, sample size, methods of lead measurement, and control of confounding factors may contribute to these discrepancies. Given the public health implications, a systematic review of the existing literature is warranted to clarify the relationship [20,21,22,23,24,25].

This systematic review aims to critically evaluate and synthesize the available evidence on the association between lead exposure in deciduous teeth and dental caries development in children, as well as the impact on the aesthetic quality of the teeth. While numerous investigations have linked lead with caries in mixed or permanent dentitions, very few have isolated the primary tooth window—a period of heightened physiological vulnerability and clinical significance.

## 2. Materials and Methods

### 2.1. Eligibility Criteria and Information Sources

This systematic review was conducted following the Preferred Reporting Items for Systematic Reviews and Meta-Analyses (PRISMA) guidelines. Inclusion criteria for this systematic review were specified as follows: (1) observational studies such as cross-sectional, case–control, and cohort designs that investigated the relationship between lead exposure and the development of dental caries in children; (2) studies that specifically involved deciduous teeth; (3) studies published in English up until April 2025; and (4) inclusion of both human participants and in vitro analyses. Exclusion criteria were the following: (1) studies that exclusively addressed permanent teeth; (2) studies that failed to report quantitative measures of both lead exposure and dental caries; and (3) studies that lacked sufficient methodological details to assess the quality of the findings.

Information sources included electronic databases such as PubMed, Scopus, and Web of Science. Additional sources encompassed manual searches of reference lists from relevant articles and gray literature to ensure comprehensive coverage. Studies focusing solely on permanent teeth or not reporting measures of lead exposure and dental caries were excluded. The systematic review was registered in the Open Science Framework with the code osf.io/8bhfr.

### 2.2. Search Strategy

A systematic search was performed using a combination of keywords and Medical Subject Headings (MeSH) terms related to lead exposure and dental caries in children. The search terms included “lead exposure”, “dental caries”, “deciduous teeth”, “primary teeth”, “children”, “blood lead levels”, “salivary lead”, “enamel defect”, “hypoplasia”, “developmental defects of enamel”, and “enamel lead levels”. Boolean operators “AND” and “OR” were used to combine terms appropriately.

The search strategy was adapted for each database to account for differences in indexing terms and search functionalities. The initial search yielded a total of 503 studies, which were screened for relevance based on titles and abstracts.

### 2.3. Study Selection and Data Extraction

Two independent reviewers screened the titles and abstracts of the identified studies to assess eligibility. Full-text articles of potentially relevant studies were retrieved for detailed evaluation. Disagreements between reviewers were resolved through discussion or consultation with a third reviewer.

Data extraction was performed using a standardized form, collecting information on study characteristics, participant demographics, methods of lead measurement, caries assessment, and key findings. The extracted data were cross-checked for accuracy and completeness by both reviewers.

### 2.4. Quality Assessment and Risk of Bias

The quality of included studies was assessed using the Newcastle–Ottawa Scale for observational studies. This tool evaluates the selection of study groups, comparability of groups, and ascertainment of exposure and outcomes. Each study was rated as low, moderate, or high risk of bias based on the scoring system. The risk of bias assessments informed the interpretation of the study findings. Studies with a high risk of bias were noted, and sensitivity analyses were considered to evaluate their impact on the overall conclusions.

### 2.5. Outcome Measures

The primary outcome measure was the association between lead exposure and dental caries in deciduous teeth, quantified through measures such as odds ratios, prevalence ratios, or correlation coefficients. Lead exposure was reported as defined by included studies. Beyond caries indices, we extracted data on developmental enamel defects (hypoplasia, opacities) when reported, as these constitute the only objective aesthetic proxy available in the included literature. Aesthetics were screened using author-defined enamel-defect or discoloration endpoints; however, only three studies reported compatible metrics, precluding quantitative pooling. Heterogeneity in exposure matrices likewise ruled out meta-analysis.

Secondary outcomes included potential mediating factors such as salivary flow rate, pH, buffer capacity, and counts of cariogenic bacteria like mutans streptococci and lactobacilli. The review also considered the presence of enamel defects and their association with lead levels.

### 2.6. Data Synthesis and Statistical Analysis

Between-study heterogeneity was to be quantified with Cochran’s Q and expressed as I^2^ and Tau^2^. Where ≥3 homogeneous effect estimates were available, we planned a DerSimonian–Laird random-effects meta-analysis. Pre-specified subgroups were blood lead, enamel lead, and saliva lead matrices.

A qualitative synthesis of the findings was conducted due to heterogeneity in study designs, measurement methods, and reported outcomes. Descriptive statistics were used to summarize the data from included studies. Where possible, quantitative data were tabulated to compare lead levels and caries outcomes across studies. Meta-analysis was not performed due to the limited number of studies and variability in methodologies. The results were organized thematically to provide a comprehensive overview of the association between lead exposure and dental caries in children.

## 3. Results

**Figure 1:**
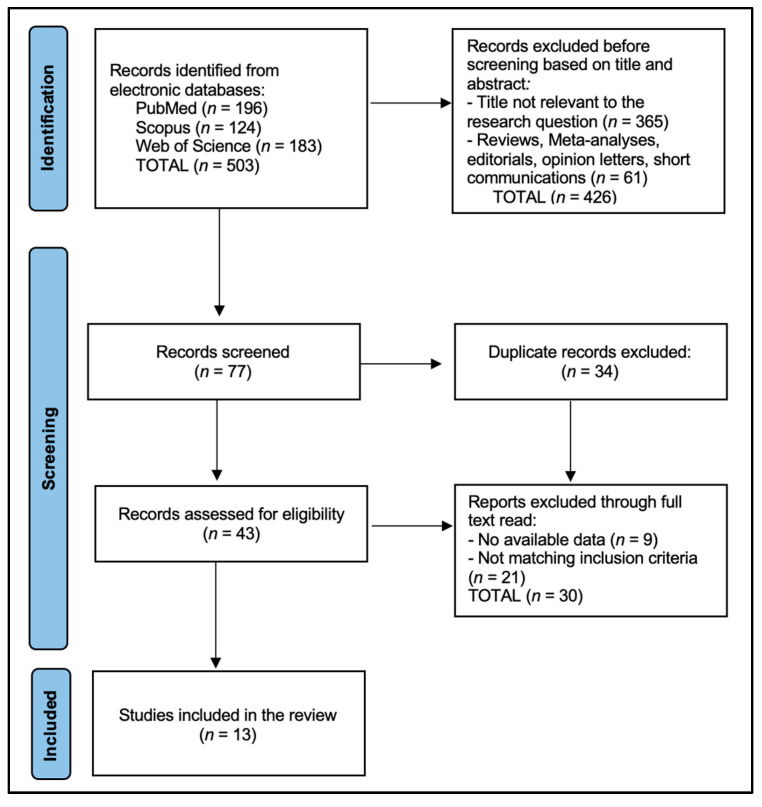
PRISMA 2020 Flow Diagram.

Of 503 records, 13 studies met inclusion criteria (Figure 1). A total of 8 used blood lead, three enamel lead, and two saliva lead assays. Table 1 presents the characteristics of the 13 included studies [14,15,16,17,18,19,20,21,22,23,24,25,26], which collectively offer a comprehensive overview of research on the association between lead exposure and dental caries in children. The studies span various countries—including Korea, Thailand, the USA, Brazil, Jordan, Canada, and Iran—providing a global perspective on the issue.

Most studies utilized a cross-sectional design, appropriate for identifying associations at a single point in time but limited in establishing causality. Notably, Campbell et al. [22] employed a retrospective cohort design, allowing for temporal analysis between early lead exposure and later caries development. The age ranges predominantly focused on children with deciduous teeth, or deciduous teeth compared with permanent teeth.

Lead exposure assessment varied across studies, including measurements of blood lead levels, salivary lead levels, lead concentrations in enamel, and lead levels in deciduous teeth using advanced techniques like inductively coupled plasma–optical emission spectrometry (ICP-OES). Caries assessments were conducted using standard indices such as dfs (decayed and filled surfaces in deciduous teeth), DMFS (decayed, missing, and filled surfaces in permanent teeth), and dmfs (decayed, missing, and filled surfaces in primary teeth). The diversity in methodologies underscores the complexity of comparing results across studies but also enriches the collective understanding of the potential relationship between lead exposure and dental caries.

Table 2 summarizes lead levels and caries outcomes, highlighting the quantitative aspects of the studies. Blood lead levels varied, with Kim et al. [14] reporting a geometric mean of 1.53 μg/dL and Youravong et al. [17] reporting a higher geometric mean of 7.2 μg/dL. Lead concentrations in teeth were reported in μg/g or ppb, with Alomary et al. [19] noting a mean lead concentration of 30.26 μg/g in deciduous teeth, while Motevasselian et al. [23] reported a mean of 213.26 ppb in primary teeth.

Caries measurements were consistent across studies, utilizing indices like dfs, DMFS, and dmfs. The majority of studies observed that higher lead levels were associated with increased caries risk or severity. For instance, Wiener et al. [16] reported adjusted mean ratios for decayed/filled teeth up to 1.94 for higher blood lead levels, indicating a strong positive association. Moss et al. [21] found that a 5 μg/dL increase in blood lead level was associated with an odds ratio of 1.8 for caries in children aged 5–17 years.

Conversely, some studies did not find a significant association. Youravong et al. [15] observed no significant differences in caries related to salivary lead levels, and Motevasselian et al. [23] reported no association between lead levels in teeth and saliva with dental caries prevalence. These discrepancies may stem from differences in lead exposure levels, the biological sample used for lead measurement, or unaccounted confounding factors.

Youravong et al. [15] assessed salivary properties and bacterial counts, finding that high salivary lead levels were associated with reduced counts of mutans streptococci, a primary bacterium involved in caries development.

Gomes et al. [18] examined the presence of enamel defects in relation to lead levels but found no association. This suggests that lead may not directly cause structural defects in enamel that could predispose to caries. Alomary et al. [19] found that tooth type and position affected metal concentrations, indicating that local factors within the oral cavity may influence lead accumulation.

Gemmel et al. [20] observed that the association between blood lead and caries was stronger in urban children, possibly due to higher environmental lead exposure in urban settings. Overall, the additional findings highlight that while there is evidence of an association between lead exposure and dental caries, the underlying mechanisms remain unclear. Factors such as salivary composition, bacterial flora, and enamel integrity may play roles, but more research is needed to elucidate these pathways.

Across blood lead studies, each 1 µg/dL increment corresponded to odds-ratio increases ranging from 1.07 to 1.25 for primary-tooth caries. Enamel lead concentrations above 75 ppm doubled dmfs in preschool cohorts, while saliva lead associations were inconsistent, likely owing to sparse sample sizes.

## 4. Discussion

### 4.1. Assessment of Findings and Additional Literature

The systematic review revealed some significant findings regarding the correlation between lead exposure and the prevalence of dental caries in children with deciduous teeth. Notably, a number of the reviewed studies showed a positive association between elevated lead levels in various biological mediums (blood, saliva, and tooth enamel) and an increased risk of dental caries. For instance, studies that measured lead concentrations directly in deciduous teeth found higher lead levels in carious compared to non-carious teeth [18,19,23,24]. This suggests that lead exposure could be a contributing factor to the development of dental caries, possibly through its effects on saliva composition and cariogenic bacteria or through direct damage to dental tissues.

From a clinical standpoint, these findings support earlier preventive recall (every 3–4 months) for children in high-lead regions and prioritization of fluoride-varnish or sealants before age 5, when primary-tooth mineralization is most susceptible.

Only Gomes et al. [18] reported enamel-defect outcomes; however, they observed no significant correlation with enamel lead; experimental studies demonstrate that Pb^2+^ can substitute for Ca^2+^ in hydroxyapatite, potentially disrupting ameloblast function. Future cohorts with photographic scoring could clarify whether subtle opacities escape current indices.

Comparing these findings with adults, in the review by Lee et al. [27], which examined 16 studies, a significant association was observed between elevated blood lead levels (PbB) and caries in deciduous teeth, where five out of six studies reported positive findings. Conversely, in permanent teeth, only three out of ten studies identified a similar association. Notably, all four studies that measured lead concentrations directly from teeth found a positive relationship with caries in both deciduous and permanent teeth. On the other hand, the study by Yepes et al. [28] analyzed data from 490 children in the ELEMENT cohort, with a mean blood lead level of 4.83 μg/dL (SD 2.2) during early childhood. Despite this exposure, no statistically significant correlation was found between these lead levels and the mean caries level, measured as decayed, missing, or filled surfaces (DMFS), which was 4.1 in adolescence.

In a study conducted by Yue Wu et al. [9], the association between lead exposure at various sensitive life periods and dental caries risks in permanent teeth was explored among 386 children living in Mexico City. The study found initial positive correlations between prenatal and early childhood lead exposure with peri-pubertal decayed, missing, and filled teeth (DMFT) scores, as exemplified by risk ratios (RR) in the unadjusted models for the second trimester (RR = 1.17 [1.00, 1.37]) and third trimester (RR = 1.20 [1.03, 1.40]). However, these associations lost statistical significance after adjusting for covariates such as sugar-sweetened beverage (SSB) intake. Interestingly, in a stratified analysis, higher peri-pubertal SSB intake amplified the relationship between lead exposure and D1MFT scores during the second (RR = 1.41 [1.06, 1.86]) and third trimesters (RR = 1.50 [1.18, 1.90]). This suggests that dietary habits may modulate the impact of lead exposure on dental health. In a similar manner, the study by Borany Tort et al. [5] reported that blood lead levels (BLL) were significantly associated with oral health problems in a cohort of 351 Korean children aged 7–15 years. Their findings highlighted significant relationships between BLL and community periodontal index (CPI), gingival index (GI), and plaque index (PI), with notably high odds ratios in the third quartile for CPI (7.21 [1.72–30.19]), GI (6.13 [1.62–23.19]), and PI (3.37 [1.10–10.34]) after adjusting for multiple confounders including socio-economic status and oral hygiene behavior.

The study by Manish Arora et al. [29] explored the relationship between long-term cumulative lead exposure and tooth loss among 333 men in the Veterans Affairs Normative Aging Study. Using a K-shell X-ray fluorescence method to measure lead concentrations in bone, the study identified that participants in the highest tertile for bone lead concentrations (tibia > 23 μg/g and patella > 36 μg/g) had significantly higher odds of extensive tooth loss (≥ 9 missing teeth), with prevalence odds ratios of 3.03 (95% CI, 1.60–5.76) for tibia lead and 2.41 (95% CI, 1.30–4.49) for patella lead, even after adjusting for confounding factors such as smoking and diabetes. In a similar manner, the study by Juan F Yepes et al. [28] examined the impact of lead exposure on dental caries within the Early Life Exposures in Mexico to Environmental Toxicants (ELEMENT) cohort of 490 children, measuring blood lead levels during ages 1–4 and assessing caries during adolescence. Despite a mean blood lead level of 4.83 μg/dL, they found no statistically significant relationship between early childhood lead exposure and dental caries later in life, suggesting that other factors like diet and oral hygiene were more influential in caries development. Nevertheless, this study was not included in the current review due to the focus on permanent teeth.

The study by H M Tvinnereim et al. [30] examined over 1200 Norwegian primary teeth and found that carious teeth exhibited significantly higher concentrations of heavy metals, with notable elevations in lead, mercury, and zinc compared to non-carious teeth. Specifically, the differences in metal concentrations between carious and non-carious teeth were statistically significant, underscoring the impact of dental caries on metal retention. Additionally, teeth with roots showed elevated levels of lead and zinc, suggesting a possible interaction between tooth morphology and metal accumulation. In a similar vein, the research by Piotr Malara et al. [31] focused on 67 impacted lower third molars and the surrounding bone from individuals in two different environmental settings. Their findings revealed markedly higher levels of cadmium and lead in both teeth and bones from individuals residing in the industrially polluted Ruda Slaska region, with cadmium levels in teeth averaging higher than those in the less polluted Bielsko-Biala region. Moreover, chromium, copper, manganese, and zinc concentrations were significantly greater in the bones surrounding the impacted teeth of those from Ruda Slaska, with specific numerical data indicating that these concentrations were not only statistically significant but also suggestive of a direct environmental influence.

Nevertheless, the historical context is essential to understand. Lead exposure has historically been a pervasive threat to human health, with children being particularly vulnerable due to both occupational and environmental factors [32]. In ancient times, the Romans made extensive use of lead, notably in their water pipelines, a practice substantiated by archaeological findings of elevated lead concentrations in skeletal remains [33]. During the pre-industrial era, children sometimes labored in lead mines, facing significant exposure to various toxic metals, while the onset of the Industrial Revolution approximately 200 years ago greatly intensified environmental contamination [34]. Over the ensuing centuries, additional sources of pediatric lead exposure have included commonplace items such as toy soldiers fashioned from lead-tin alloys, which could leach lead upon handling or mouthing [32,34]. Chronic exposure to lead has been linked not only to systemic toxicity but also to an increased risk of dental caries and disrupted tooth development, underscoring the importance of preventive measures throughout history and into the present day [21].

The novelty of this study lies in conducting a systematic review that not only delivers a higher level of evidence but also uniquely compiles the most extensive collection of studies specifically focusing on the development of caries due to lead exposure in children and adolescents. The implications of this study also extend to dental aesthetics, a critical component of pediatric dentistry that can influence a child’s self-esteem and social interactions. Understanding the role of lead exposure in the development of dental caries and potentially in the formation of enamel defects, which were not conclusively linked in this review but are commonly associated with aesthetic concerns, can help in planning aesthetic restorations. Treatment planning for children exposed to lead might need to consider the potential for increased caries risk and enamel fragility, guiding the choice of more durable and protective restorative materials. Additionally, public health measures aimed at reducing lead exposure in children could help decrease the prevalence of dental caries and associated aesthetic issues, thus contributing to better overall oral health outcomes and enhanced quality of life for affected individuals.

### 4.2. Study Limitations

With just one cohort among 13 studies, temporality remains unresolved; longitudinal designs are essential to disentangle exposure timing from caries onset. Study heterogeneity—spanning three distinct lead matrices, four caries indices (dmfs, dfs, DMFS, DMFT), and mostly cross-sectional designs—precludes pooling and weakens causal inference. Differential limit-of-detection thresholds further complicate comparability. Funnel-plot inspection for publication bias was impossible, but the risk remains. Therefore, the review’s ability to draw definitive conclusions is limited by the variation in study methodologies and the lack of uniformity in measuring lead exposure and dental caries outcomes. The inclusion of only observational studies, predominantly cross-sectional, poses challenges in establishing causality between lead exposure and dental caries. Furthermore, potential confounding factors, such as socio-economic status, dietary habits, and oral hygiene practices, which could influence both lead exposure and caries risk, were not uniformly accounted for across the studies. Finally, the variability in lead measurement techniques and the reliance on self-reported data in some studies could introduce measurement bias, affecting the accuracy of the reported association.

## 5. Conclusions

The findings of this systematic review suggest a complex and nuanced relationship between lead exposure in deciduous teeth and the development of dental caries in children. While several studies indicate a positive association between higher lead levels and increased caries risk, this link is not uniformly observed across all included studies. Significant variability in lead levels and dental caries outcomes points to the potential influence of environmental and biological factors, as well as the methodological diversity among studies. Although the evidence supports concerns about lead exposure as a risk factor for dental caries, it also highlights the need for further research to clarify the mechanisms involved and the influence of mediating factors like enamel integrity and cariogenic bacteria.

## Figures and Tables

**Table 1:** Overview of Characteristics and Key Findings of Studies Assessing the Impact of Lead Exposure on Dental Caries.

Authors	Year	Country	Study Design	Sample Size	Age Range	Lead Measurement	Caries Assessment	Objectives	Quality Assessment and Risk of Bias
Moss et al. [21]	1999	USA	Cross-sectional	24,901	≥2 years	Blood lead levels	Decayed and filled surfaces	Blood lead level significantly associated with number of carious surfaces; higher blood lead levels increased risk of dental caries	Score: 8/9 Bias: Low
Campbell et al. [22]	2000	USA	Retrospective cohort	248	Second & fifth graders	Blood lead levels (mean between 18–37 months)	DMFS (permanent), dfs (deciduous)	Lead exposure (>10 μg/dL) not a strong predictor of caries; adjusted odds ratio not significant	Score: 6/9 Bias: Moderate
Gemmel et al. [20]	2002	USA	Cross-sectional	543	6–10 years	Blood lead levels	Number of carious tooth surfaces	Blood lead levels associated with caries among urban children; stronger association in primary teeth	Score: 6/9 Bias: Moderate
Gomes et al. [18]	2004	Brazil	Cross-sectional	329	Preschool children	Lead concentration in enamel	dmfs index	Higher proportion with dental caries among those with higher lead concentrations in deciduous teeth (non-industrial area); no association with enamel defects	Score: 5/9 Bias: Moderate
Youravong et al. [17]	2006	Thailand	Cross-sectional	292	6–11 years	Blood lead levels (PbB)	dfs (deciduous), DMFS (permanent)	Cariogenicity of lead evident in deciduous teeth but not in permanent teeth for this age group	Score: 6/9 Bias: Moderate
Martin et al. [26]	2006	USA	Cross-sectional	507	8–12 years	Blood lead levels	DMFT index	Gender-specific association between lead exposure and dental caries in primary teeth only (males)	Score: 6/9 Bias: Moderate
Youravong et al. [15]	2013	Thailand	Cross-sectional	120	Not specified	Salivary lead levels	dfs (deciduous), DMFS (permanent)	No association between salivary lead levels and dfs or DMFS; salivary lead levels correlated with blood lead levels	Score: 5/9 Bias: Moderate
Alomary et al. [19]	2013	Jordan	Cross-sectional	320	5–12 years	Lead levels in deciduous teeth (ICP-OES)	Caries status (presence of caries)	Relation between metal concentrations in teeth and caries status; higher lead levels in carious teeth	Score: 6/9 Bias: Moderate
Pradeep et al. [24]	2013	India	Cross-sectional	90	5 years	Enamel and salivary lead levels assessed by graphite atomic absorption spectrophotometry	dmfs index	Increased lead levels in enamel and saliva were associated with higher severity of dental caries. A positive correlation between enamel and salivary lead levels was observed. No gender difference in lead accumulation.	Score: 4/9 Bias: High
Wiener et al. [16]	2014	USA	Cross-sectional	3127	24–72 months	Blood lead levels	Number of decayed/filled teeth	Strong association between blood lead levels and increasing numbers of carious teeth	Score: 6/9 Bias: Moderate
Kim et al. [14]	2017	Korea	Cross-sectional	1564 (permanent), 1241 (deciduous)	School-aged	Blood lead levels	Decayed and filled surfaces (dfs)	Increased risk of dental caries in deciduous teeth with higher blood lead levels (<5 μg/dL)	Score: 8/9 Bias: Low
Sanders et al. [25]	2019	USA	Cross-sectional	12,373	2–19 years	Blood lead levels	dmfs	Non-consumers of tap water had lower prevalence of elevated blood lead levels and higher prevalence of dental caries	Score: 7/9 Bias: Moderate
Motevasselian et al. [23]	2023	Iran	Cross-sectional	211	6–11 years	Lead and cadmium levels in primary teeth and saliva	Dental caries prevalence	No association between Pb and Cd concentrations in primary teeth and saliva with dental caries prevalence	Score: 5/9 Bias: Moderate

dfs—Decayed and Filled Surfaces; DMFS—Decayed, Missing, and Filled Surfaces (permanent teeth); dmfs—Decayed, Missing, and Filled Surfaces (deciduous teeth); PbB—Blood Lead Levels; ICP-OES—Inductively Coupled Plasma Optical Emission Spectrometry; PR—Prevalence Ratio; CI—Confidence Interval; OR—Odds Ratio; SD—Standard Deviation; Rs—Spearman’s Rank Correlation Coefficient.

**Table 2:** Summary of Lead Exposure Levels and Dental Caries Outcomes Across Included Studies.

Authors	Lead Levels Measured	Mean Lead Levels	Caries Measurement	Caries Outcomes	Conclusions
Moss et al. [21]	Blood lead levels	Significant log of blood lead levels	Decayed and filled surfaces	Among children 5–17 yrs, a 5 μg/dL increase in Pb associated with OR 1.8 (95% CI: 1.3–2.5) for caries	Lead exposure may explain income and regional differences in caries prevalence
Campbell et al. [22]	Blood lead levels	Categories: <2 μg/dL, 2–5 μg/dL, 5–10 μg/dL, >10 μg/dL	Number of decayed/filled teeth	Adjusted mean ratios: 1.79 (2–5 μg/dL), 1.88 (5–10 μg/dL), 1.94 (>10 μg/dL)	N/A
Gemmel et al. [20]	Blood lead levels	Mean 2.3 μg/dL (SD 1.7); urban mean 2.9 μg/dL, rural mean 1.7 μg/dL	Number of carious tooth surfaces	Positive association in urban children (*p* = 0.02); stronger in primary teeth	Association between blood lead and caries stronger in urban children
Gomes et al. [18]	Lead concentration in enamel	Obtained via enamel biopsy	dmfs index	Higher caries prevalence among those with higher enamel lead levels (non-industrial area)	No relationship between lead and enamel defects
Youravong et al. [17]	Blood lead levels (PbB)	Geometric mean 7.2 μg/dL (SD 1.5 μg/dL)	dfs (deciduous), DMFS (permanent)	Correlation between dfs and PbB: Rs = 0.25, *p* = 0.00; Adjusted OR for dfs > 5: 2.39 (95% CI: 1.36–4.20)	Lead exposure associated with caries in deciduous teeth
Martin et al. [26]	Blood lead levels	Median BLLs were 2.9 μg/dL for children aged 8–12 years	DMFT index	Found significant caries in primary teeth only for males (16.7 carious surfaces in male vs. 14.6 in female, *p* < 0.05)	No significant correlation between BLLs and caries in permanent teeth or for females in either dentition
Youravong et al. [15]	Salivary lead levels	Mean 2.26 μg/dL (range 0.16–28.52 μg/dL)	dfs and DMFS	No significant differences in caries related to salivary lead levels	High salivary Pb associated with reduced mutans streptococci counts
Alomary et al. [19]	Lead levels in deciduous teeth (ICP-OES)	Mean Pb: 30.26 μg/g	Caries status (presence of caries)	Significant differences in metal concentrations between carious and non-carious teeth	Pb levels not significantly different due to sex; Pb decreased at age 11–12
Pradeep et al. [24]	Enamel and salivary lead levels	Enamel: 47.7 ppm (Control), 85.45 ppm (ECC), 90.43 ppm (S-ECC); Saliva: 0.23 ppm (Control), 1.7 ppm (ECC), 1.77 ppm (S-ECC)	dmfs index	Increase in mean enamel lead levels from Control (47.7 ppm) to ECC (85.45 ppm) and to S-ECC (90.43 ppm) was statistically very highly significant (*p* < 0.001). Small increase from ECC to S-ECC not significant (*p* = 0.114).	No significant gender differences in lead levels; enamel (Male: 75.53 ppm, Female: 73.52 ppm, *p* = 0.812), saliva (Male: 1.30 ppm, Female: 1.17 ppm, *p* = 0.413). Correlation between enamel and salivary lead levels showed no significant differences among groups (*p* > 0.05).
Wiener et al. [16]	Blood lead levels	Categories: <10 μg/dL vs. ≥10 μg/dL	DMFS (permanent), dfs (deciduous)	Adjusted OR for dfs ≥ 1: 1.77 (95% CI: 0.97–3.24; *p* = 0.07)	Results should be interpreted cautiously due to limitations
Kim et al. [14]	Blood lead levels	Geometric mean 1.53 μg/dL (max 4.89 μg/dL); 74.4% had <2 μg/dL	Decayed and filled surfaces (dfs)	Children with caries had higher mean blood lead (1.59 μg/dL) than those without (1.51 μg/dL)	N/A
Sanders et al. [25]	Blood lead levels	3% had elevated levels ≥3 μg/dL	DMFT index	50% of children had dental caries experience	Lower prevalence of elevated BLL in non-tap water consumers (Adjusted PR = 0.62, 95% CL = 0.42, 0.90)
Motevasselian et al. [23]	Lead and cadmium levels in teeth and saliva	Teeth Pb mean: 213.26 ppb; Saliva Pb mean: 11.83 ppb	Dental caries prevalence	No association between Pb and Cd concentrations in primary teeth and saliva with dental caries prevalence	Pb and Cd in teeth and saliva not associated with socioeconomic status, oral hygiene, or snacking frequency

dfs—Decayed and Filled Surfaces; DMFS—Decayed, Missing, and Filled Surfaces (permanent teeth); dmfs—Decayed, Missing, and Filled Surfaces (deciduous teeth); PbB—Blood Lead Levels; ICP-OES—Inductively Coupled Plasma Optical Emission Spectrometry; PR—Prevalence Ratio; CI—Confidence Interval; OR—Odds Ratio; SD—Standard Deviation; Rs—Spearman’s Rank Correlation Coefficient.

## Data Availability

Not applicable.
